# An Integrated Portable Multiplex Microchip Device for Fingerprinting Chemical Warfare Agents

**DOI:** 10.3390/mi10090617

**Published:** 2019-09-16

**Authors:** Karolina Petkovic, Anthony Swallow, Robert Stewart, Yuan Gao, Sheng Li, Fiona Glenn, Januar Gotama, Mel Dell’Olio, Michael Best, Justin Doward, Simon Ovendon, Yonggang Zhu

**Affiliations:** 1 CSIRO Manufacturing, Bayview Ave, Clayton 3168, Australia; 2 DST, 506 Lorimer Street, Fishermans Bend, VIC 3207, Australia; 3 Harbin Institute of Technology (Shenzhen), Shenzhen, Guangdong 518055, China; 4 School of Science, RMIT University, Melbourne, VIC 3001, Australia

**Keywords:** lab on a chip, chemical warfare agent, conductivity sensor, microchip capillary electrophoresis

## Abstract

The rapid and reliable detection of chemical and biological agents in the field is important for many applications such as national security, environmental monitoring, infectious diseases screening, and so on. Current commercially available devices may suffer from low field deployability, specificity, and reproducibility, as well as a high false alarm rate. This paper reports the development of a portable lab-on-a-chip device that could address these issues. The device integrates a polymer multiplexed microchip system, a contactless conductivity detector, a data acquisition and signal processing system, and a graphic/user interface. The samples are pre-treated by an on-chip capillary electrophoresis system. The separated analytes are detected by conductivity-based microsensors. Extensive studies are carried out to achieve satisfactory reproducibility of the microchip system. Chemical warfare agents soman (GD), sarin (GB), O-ethyl S-[2-diisoproylaminoethyl] methylphsophonothioate (VX), and their degradation products have been tested on the device. It was demonstrated that the device can fingerprint the tested chemical warfare agents. In addition, the detection of ricin and metal ions in water samples was demonstrated. Such a device could be used for the rapid and sensitive on-site detection of both chemical and biological agents in the future.

## 1. Introduction

Lab-on-a-chip (LOC) devices have the potential to revolutionize modern medicine, environmental monitoring, and a range of industrial applications due to their advantages such as portability, rapid analysis, automation, and reduced usage of sample and reagents [1,2,3,4,5]. The research in the field has exploded over the last 20 years, resulting in numerous applications in medicine [6,7,8], food security [9], and environmental monitoring [10,11,12]. Chemical and biological warfare agents have been the subject of significant scientific research over the last 20 years due to the increased threat of terrorism and usage in war zones. Many detection techniques have been developed to identify chemical warfare agents (CWA) such as G-type and V-type nerve agents. Alkylphosphonic acids, the nerve agents’ hydrolysis degradation products, are specific to their parent nerve agents, and can also be used for such identifications in environmental samples [13]. The developed detection techniques include infrared spectroscopy, ion mobility spectroscopy, capillary electrophoresis (CE), GC-MS, and LC-MS [14,15].

CE has emerged as a suitable analytical method for the identification of nerve agents [16,17,18]. This technique has been extensively studied for applications in chemical and biological analysis [19,20,21,22,23,24,25,26]. The prevailing CE-based detection techniques for CWAs are UV [27,28,29] and conductivity detection. Of all the aforementioned techniques, the microfluidics-based LOC concept is the most attractive for identification of chemical and biological warfare agents in the field due to its inherent potential for the integration of filtration, preconcentration, separation, and detection on one platform [16,30,31,32,33,34,35,36]. Microchip capillary electrophoresis (MCE) was the electrophoretic sample separation method adopted for LOC devices, and the capacitively coupled contactless conductivity detection (C^4^D) was the detection method of choice. With small injection volumes, short separation channels, and a high electric field across them, MCE is designed to result in analysis times of several seconds. In addition, it enables low sample and reagents consumption and higher integration into portable devices. C^4^D is based on two electrodes arranged along a conduit containing an electrolytes-based sample solution. The electrodes form capacitance in an electric double layer of the microchannel that allows the passage of alternating currents. C^4^D is an attractive proposition due to its low power requirements and low cost of miniaturization and integration. C^4^D was initially introduced by coupling with CE [37,38,39], and later with MCE [30,31,32,36,40,41,42].

A useful and commercially attractive analytical instrument for a quick and accurate detection of chemical warfare agents (CWA) in environmental samples has to satisfy several criteria such as portability, sample processing, sensitivity, selectivity, reproducibility, analysis time, low false alarms, power requirements, ongoing cost, simplicity of use, and safety of operation [43,44]. The main challenge for currently commercially available portable instruments for the detection of CBW agents is to increase the reproducibility, sensitivity, and specificity; and to reduce the frequency of false alarms operation. Hauser at al. was the first to develop a prototype device based on the concept of a portable CE [24]. Kuban et al. developed a portable CE-C^4^D instrument for an in situ analysis of sarin (GB), soman (GD), and VX in environmental samples [43]. Internal standards were used to improve the reproducibility of analytical results. Including sample preparation, results were generated in 10 minutes. However, the instrument was not tested for false alarms, and no information was available about the user interface, power consumption, and ongoing costs. In addition, it did not have multiplexing capability. Saiz et al. developed a portable CE-C^4^D instrument for the determination of nitrogen mustard (HN) in water samples [45]. The problem of reproducibility was addressed by optimizing different capillary coating procedures. The limit of detection was down to 5 µM. A CE-C^4^D-based mobile platform for air sampling has been developed by da Costa et al. [46]. The system could not be tested on real HN, but it has demonstrated its potential for the remote sensing of organic acids in air samples [46]. Ansari et al. developed a modular, MCE-C^4^D-based and battery-powered instrument with improved dual top-bottomC^4^D [47]. The instrument was tested on inorganic cations, anions, and urine and blood samples with the limit of detection of 5 µM. The instrument has a potential to be used for determination of chemical and biological agents.

The aim of this study is to develop an integrated LOC system for CWA detection in the field. To the best of our knowledge, this paper presents the first portable, multiplex, MCE-C^4^D device for the on-field detection of CWA agents and addresses several critical issues hindering the commercial acceptance of LOC technology. These include the batch-to-batch reproducibility of the microchip separation, an accuracy of detection in environmental samples with interference substances, cost and speed per analysis, lack of testing data from real warfare agent samples, and its associated device design considerations.

## 2. Methodology

The methodology of the current device is to detect the presence of chemical warfare agents together with their degradation products. In realistic scenarios, chemical agents and their degradation products may co-exist, thus providing a unique signature of detection that minimizes the false alarm rate. For the chemical warfare agents soman (GB), sarin (GD), and VX, the hydrolysis products are pinacolyl methylphosphonic acid (PMPA), isopropyl-methylphosphonic acid (IMPA), and ethyl methylphosphonic acid (EMPA), respectively. These products will be further degraded slowly over time to methylphosphonic acid (MPA) [48]. We use a MCE-based molecule separation as a pre-treatment step to the sample and use a C^4^D sensor to detect the presence and concentration of these chemical substances. The aim is to provide a unique signature of detection in environmental samples and to reduce the rate of the false alarms.

## 3. Experimental Details

### 3.1. Chemicals

All chemicals employed in this study were of analytical grade, and ultra-pure water (Milli-Q systems, 18.2 MΩ × cm (mega-ohms) at 25 °C) were used throughout. Methylphosphonic acid (MPA) and its sodium salts: ethyl methylphosphonic acid (EMPA) and pinacolyl methylphosphonic acid (PMPA) were purchased from Sigma Aldrich, while isopropyl methylphosphonic acid (IMPA) was obtained from Cerilliant Corporation (Austin, TX, USA). The background electrolyte (BGE) (10 mM MES/His, pH = 5.9) for microchip electrophoresis measurements was prepared daily from stock solutions of 2-(*N*-morpholino) ethanesulfonic acid (MES), DL-histidine (His), and 3-morpholino-2-hydroxypropane sulfonic acid (MOPSO), which were obtained from Sigma Aldrich. The environmental samples were collected from the Yarra River (Melbourne, Australia). Standards of GB, GD, VX, and ricin samples were supplied and handled by the staff of the Defence Science and Technology (DST, Melbourne, Australia). All measurement tests involving the real chemical warfare agents were conducted at DST laboratories.

### 3.2. Microchip Fabrication

**Figure 1:**
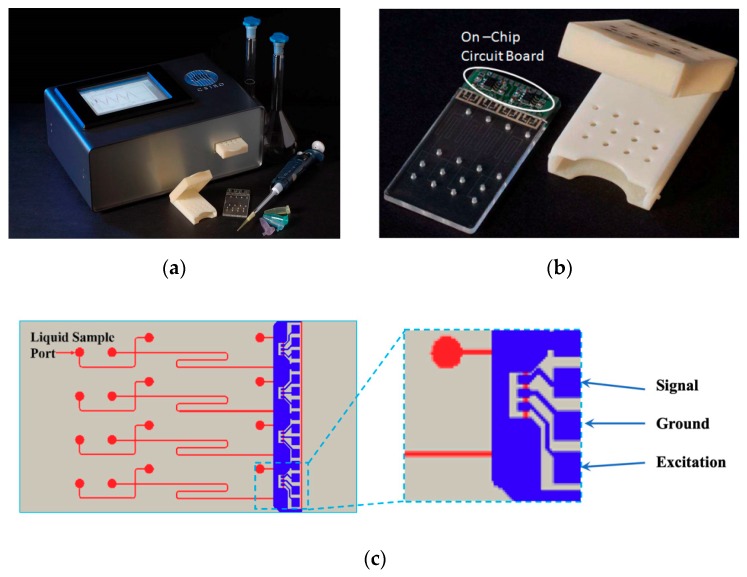
The prototyped instrument: (**a**) The main integrated instrument; (**b**) Four-channel microchip with integrated front-end electronics and housing cartridge; (**c**) Sketch of a four-channel microchip and contactless conductivity detection method.

A high-resolution transparency mask (2400–20000 dpi) was used in the transfer of the four-channel, simple T-cross and polycarbonate-based, microfluidics chip (70 µm × 70 µm, channel’s cross-section) pattern onto a dry laminar resist (Shipley 5038, Shioley, Marlborough,m MA, USA) by UV lithography (Collimated UV exposure system, ABM-USA Inc, San Jose, CA, USA) (Figure 1c). The four-channel design was adopted to enable multiplexing and simultaneous detection to achieve a high throughput of detection. 

A sputter deposition of a thin film of nickel (100 nm) and electroplating of 200 µm of nickel (Ni sulfamate bath, 45 °C, 20 mA/${\mathrm{cm}}^{2}$) followed in the process of the nickel shim fabrication. The nickel shim, whose design is equivalent to the design of the four-channel microchip depicted in Figure 1c, was used in the process of injection molding for fast microfluidics chips prototyping (Battenfeld BA800 CDC, Battenfeld, Awans, Belgium). The 2-mm diameter liquid reservoirs (Figure 1c) were drilled and 150 µm of PET (poly(ethylene terephthalate)) film was used to thermally seal the microfluidics chips.

### 3.3. C^4^D Sensor Fabrication

A four-$C^{4}D$, planar sensor array pattern, was deposited on the microchip PET capping layer using a stencil mask, metal evaporation, and the deposition from a resistive boat. The deposition of Cr (15 nm) and Ag (150 nm) was performed at 5 × 10^−5^ Torr. The $C^{4}D$ detection cell geometry adopted in this work has been described in detail previously [49,50,51]. The PET film was an insulating layer between the deposited C^4^D electrodes and the liquid inside the channels and allowed for the capacitive coupling between the two.

### 3.4. Microchip Electrophoresis

All microchip electrophoresis tests were carried out starting with the same preconditioning protocol. The protocol consisted of flushing the microfluidics channels with 1 M NaOH solution for 10 min, followed by rinsing with Millipore water for 10 min and finally conditioning with the BGE solution for 10 min. The strict application of this protocol was critical for obtaining the desired reproducibility of analytical results. The other critical point was a precise dispensing of the same amount of liquid in the liquid reservoir to avoid the effects of induced pressure-driven flow. The sample injection was accomplished by applying an electric field across the sample injection channel, while the separation was accomplished by applying an electric field across the separation channel.

## 4. Device Development

### 4.1. Development of Portable Multiplex Microfluidics Device

A prototype, portable, multiplex microchip instrument for the on-field detection of the chemical warfare agents with dimensions of 200 × 100 × 50 mm (length × width × height) that is powered by a 12 V DC power adapter is presented in Figure 1a. The grounded instrument main body is housing three main components: a microfluidics module coupled to a $C^{4}D$ detection circuit board, a high-voltage (HV) power supply module, and a tablet PC with a touch screen for the data analysis and display.

### 4.2. Microfluidics and High Voltage Power Supply Module

The HV power supply module consists of four small EMCO C-series high voltage modules (XP Power, Sunnyvale, CA, USA) and switching relays interfaced to the microfluidics module via 16 HV cables (four per each microfluidic channel). These HV power supply units were selected because of their high filtered output, a reduced noise on the high voltage rails, and because an embedded controller can easily control them. The maximum output voltage was 5 kV. Each power supply uses an ATmega 8-16 microcontroller (Microchip Tech. Inc., Chandler, Arizona, USA) to program the electrophoresis procedures electric field requirements and the time needed for the test runs. In addition, the controllers were monitoring the DC current in both injection and separation microchannels. The four high voltage circuits are coordinated over an I2C communications bus by one master controller, which handles all the data interfacing with the main touch-panel display unit. A rapid prototyping technique was adopted for the fabrication of the main microfluidics housing body and the HV connection plate. A set of 16 sprung connectors, mounted at 30° from vertical on the HV connection plate, and wired to the HV cables was used to make the high-voltage contact with the copper discs on the lid of the cartridge. A signal conditioning circuit was secured on the rear of the main housing body, and interfaced to a data acquisition board (USB-6210, 10v max, National Instrument, Austin, Texas, USA) for analog to digital processing. It provided a signal DC offset, amplification, and active rectification.

To increase the signal and reduce the noise, the four dual operational amplifiers (OPA21132) with a high bandwidth and low offset voltage are mounted on a circuit board close to the $C^{4}D$ sensor array on the chip. The board had an extra ground plane to provide the shielding from the AC excitation signal (XR2206, 14$\;V_{PP}$ 100–380 kHz), and it was connected to the sensor array by means of fine wires and conducting epoxy, which provided a very good physical and electrical connection. The main purpose of the chip cartridge (Figure 1b) is to avoid the contamination of the instrument. Together with the polycarbonate, the microchip is designed as a single use, disposable unit. In addition, it holds the copper discs for interfacing with the HV cables. The platinum electrodes (500 µm) were incorporated to the copper discs on the hinged lid to coincide with the microchip liquid reservoirs. When the chip and the chip cartridge are inserted into the main housing body, the buffered sensor signal is passed to a signal conditioning circuit at the rear of the housing for rectification, processed by a data acquisition card, and sent to the PC on board for further processing and displaying.

### 4.3. Display Module and Graphic User Interface (GUI)

The data analysis and display module embodies an industrial panel PC (PPC-L62T, Advantech, Milpitas, CA, USA). The two primary applications are intended for the PC. The first application is for the laboratory testing purposes as a mean for developing libraries for CWAs of interest and their relative interferents. This application allows the expert user a high-level control over the device and the real-time data viewing. The setup page of the user interface allows the definition of electrophoretic operational conditions for each of four channels on a microfluidics chip. The second application is for on-field use and provides a high level of abstraction for an easy on-field operation. Accordingly, it features a single large push button, which covers the upper half of the display for the start/stop operation while wearing heavy protective gear and thick gloves. The PC interfaces to the data acquisition board via a USB link and to the device electronics via an RS232 serial link. Commands sent via the RS232 are received by a master microcontroller, which uses a two-wire serial interface (TWI) to control multiple slave microcontrollers. In turns, each slave controls a single channel pairing on the microfluidics chip by providing a pulse-width modulation (PWM) to control high-voltage modules, switching relays, and sampling current sensor readings.

## 5. Results

### 5.1. Designing Background Electrolyte for MCE-C^4^D

To maximize the sensitivity of the conductivity detection, a higher mobility of the BGE counter-ion and a higher difference between the mobilities of a BGE co-ion and an analyte ion are desired. At the same time, this difference should be minimal to satisfy the basic criteria of the capillary electrophoresis, as this difference usually leads to a peak shape deformation (fronting or tailing) [39]. Hence, designing a BGE composition for MCE-C^4^D is not a trivial task, and it is usually performed experimentally. Following the Plackett–Burman experimental design method, and by testing several co-ions (MES, MOPSO), counter-ions (His, Arg, Tris), electroosmotic flow modifiers (TTAB (tetradecyltrimethylammonium bromide), CTAB (cetyltrimethylammonium bromide)), the BGE selected for the detection of warfare agent degradation products in this study was 10 mM MES/His and 10 µM CTAB (pH = 5.9, σ = 256 µS/cm). The same BGE composition was used previously for the detection of CWA degradation products by capillary electrophoresis [27,52]. The CTAB, a cationic surfactant, was used as an electromosis flow (EOF) reverser for the detection of anions. A large system peak originated from bromide and as previously reported [52], was not observed. This was probably due to the low concentration of CTAB, which was sufficient to reverse the EOF on the polycarbonate microchannel surface. Pumera et.al [16] reported that polymethylmethacrylate (PMMA)-based microchips did not require an EOF reverser due to the low EOF on their native surface. This study confined similar findings for PC surfaces, but adopted the use of CTAB and a glycerol additive to improve the reproducibility of the analytical results. The significant advantage of the instrument presented here when compared to CE-based portable devices is its multiplexing capability. The BGE composition requirements will be different for different types and mixtures of environmental samples contaminated with CWA. This instrument offers the possibility of screening the four different BGE combinations in one test run and reducing the time on the field necessary to find the optimum separation conditions.

### 5.2. Reproducibility, Linearity and Limit of Detection

While performing routine test runs on the instrument presented here, irreproducible migration times and the peaks’ shapes were observed. A number of different, interconnected factors were identified as a likely cause; out of these, the Laplace pressure-induced volumetric flow rate, superimposing to the EOF, was identified as the major contributor. Increasing the BGE viscosity was identified as one way of reducing the effects of Laplace pressure on the reproducibility of data in MCE-$C^{4}D$. The reproducibility, linearity, and limit of detection (LOD) data for the determination of NA degradation products using a modified, higher viscosity, BGE (10 mM MES/His, 10 µM CTAB 30% glycerol) are presented in Table 1.

**Figure 2:**
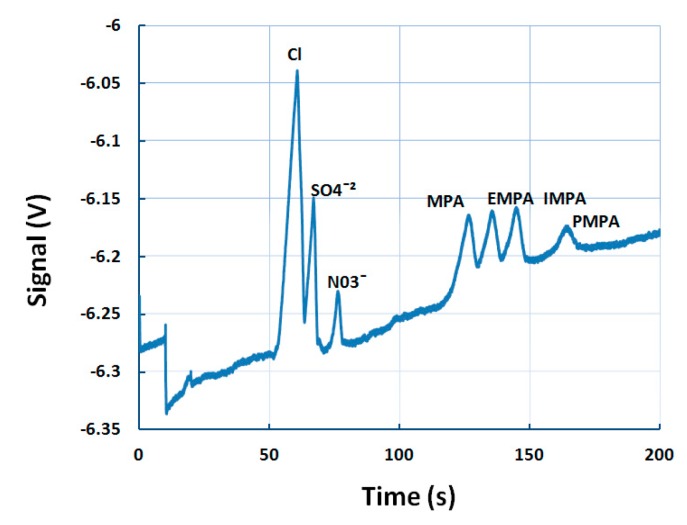
An electropherogram of an anionic mixture (100 µM ${\mathrm{Cl}}^{-}$, ${\mathrm{SO}}_{4}^{2-}$, and ${\mathrm{NO}}_{3}^{-}$, 50 ppm MPA, EMPA, IMPA, PMPA). BGE was 10 mM MES/His, 10 µM CTAB, 30% glycerol. Sample injection: 10 s, 1 kV, sample separation: 200 s, 3 kV. BGE: background electrolyte, MES: 2-(*N*-morpholino) ethanesulfonic acid.

Ten four-channel, polycarbonate microchips were randomly selected from a batch of 50. Three consecutive runs were performed on each microchip, resulting in 120 test runs in total. The sample solution consisted of 50 µM of MPA, IMPA, EMPA, and PMPA; and 200 µM of ${\mathrm{NO}}_{3}^{-}$nitrate). Nitrate was used as an internal standard to calculate the relative mobility of each analyte. By dividing the analyte migration time with the nitrate migration time, a very good relative standard deviation (RSD, %) was obtained ranging from 0.5% for MPA up to 1.1% for EMPA. Only PMPA showed a slightly higher value of 2.6%. These results were comparable or higher from the same reported for a CE portable instrument [18], and MCE-$C^{4}D$ [19]. The linearity of the $C^{4}D$ response was tested on different concentration solutions ranging from 50 µM to 200 µM. For all four degradation products, the linearity correlation coefficient was equal or higher than 0.98. The limits of detection, calculated as three times the median noise level (3 mV), are 1.73 ppm, 3.73 ppm, 3.73 ppm, and 4.13 ppm for MPA, EMPA, IMPA, and PMPA, respectively. PMPA was the worst performing in LOD and reproducibility tests, but had the best linearity correlation coefficient. In environmental samples, CWA degradation products will be mixed with other analytes, such as inorganic anions. Figure 2 depicts the separation of an anionic mixture comprising of chloride, sulfate, nitrate, MPA, EMPA, IMPA, and PMPA. The relative migration times were 1.76 s, 1.90 s, 2.04 s, and 2.32 s for MPA, EMPA, IMPA, and PMPA, respectively. Similar work was carried out previously, where CWA degradation products were separated in the Rio Grande water [16]. In comparison, that study reported the same separation time of 150 s for the degradation products, but the separation of, ${\mathrm{NO}}_{3}^{-}$, and ${\mathrm{SO}}_{4}^{2-}$ was not achieved. That was probably due to a higher degree of sample axial diffusion along the PMMA channel surface.

### 5.3. Detection of GB and VX in Environmental Water Samples

**Figure 3:**
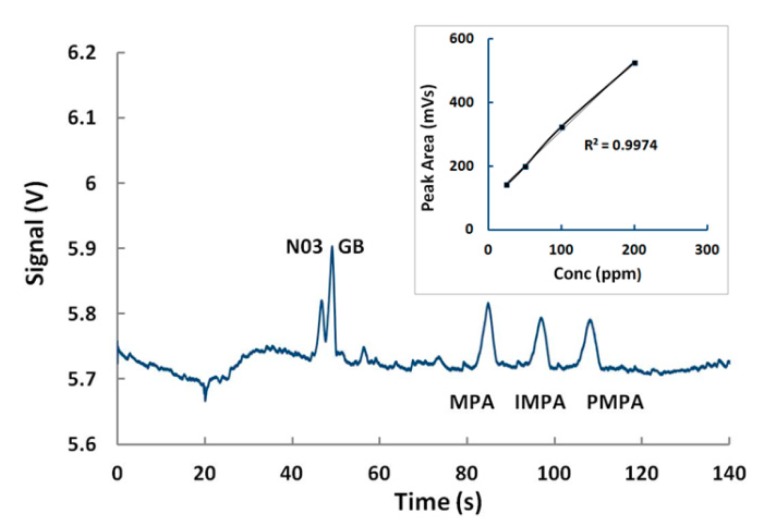
Fingerprinting of sarin and linearity of detection. Sample (100 µM N03, 50 ppm GB, MPA, IMPA, PMPA). BGE was 10 mM MES/His, 10 µM CTAB, 0.5% agarose. Sample injection: 20 s, 1 kV, sample separation: 200 s, 3 kV. CTAB: cetyltrimethylammonium bromide. GB: sarin.

For the detection of nerve agents, beside glycerol, agarose was tested as a possible “viscosity additive” for MCE-$C^{4}D$. A range of concentrations was investigated (0.5%–1%), and the concentration of 0.5% was selected as the best performing. By adding 0.5% of agarose in 10 mM MES/His and 10 µM CTAB, GB was reproducible, which was detected together with its degradation products (IMPA, MPA) in less than 80 s (1.5% RSD) (Figure 3). The calibration curve for neat GB peak areas was linear, with an excellent coefficient of variation $R^{2}=0.99$ (Figure 3 inset). The LOD was experimentally established to be approximately 500 ppb.

**Figure 4:**
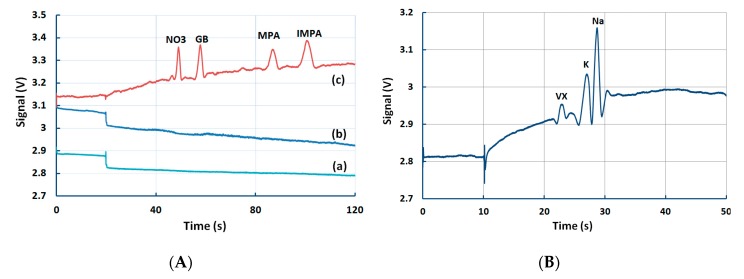
Detection of GB and VX in spiked samples (**A**) GB in spiked tap water sample (100 ppm GB, 50 ppm MPA, IMPA). BGE: 10 mM MES/His, 10 µM CTAB, 0.5% agarose. CE conditions: injection: 20 s, 1 kV, sample separation: 200 s, 3 kV. (**B**) VX in spiked Yarra River water sample (100 ppm VX). BGE was 10 mM MOPS + 30 mM Arg (pH = 7.5). CE conditions: injection: 10 s, 1 kV, sample separation: 200 s, 3 kV. CE: capillary electrophoresis.

The current device has also demonstrated the ability to detect the presence of CWAs in spiked water samples. Figure 4 shows the detection of GB in spiked tap water and VX in spiked Yarra River (Melbourne, Victoria). All water samples were mixed with 100 mM MES/His (1:10) to maintain a pH value of 5.9. The tap water was spiked with 100 ppm GB, and 50 ppm MPA and IMPA, and the three tests were performed in parallel (Figure 4A). The BGE (Figure 4A(a)) and the raw tap water sample (Figure 4A(b)) had no significant peaks, while in comparison, the spiked sample showed the fingerprinting of GB in the tap water (Figure 4A(c)). In a separate test, the Yarra River water was spiked with VX only (Figure 4B). VX could not be detected in anodic mode, but was detected in cathodic mode using a different BGE (10 mM MOPS (3-(N-morpholino)propanesulfonic acid) and 30 mM Arg (pH = 7.5)). The presence of K and Na was also confirmed.

**Figure 5:**
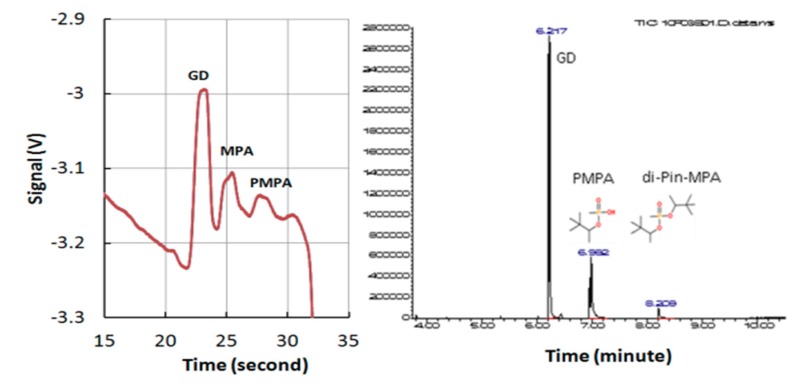
Detection of soman (GD) using the current device and its comparison with GC results. Left: results from current device, Right: results from a commercial GC-MS device. Sample (100 ppm GD, MPA, PMPA). BGE, 10 mM MOPSO, 30 mM Arg, 10 µM CTAB, 30% methanol. CE conditions: injection: 10 s, 1 kV, sample separation: 200 s, 3 kV. MOPSO: hydroxypropane sulfonic acid.

To further demonstrate the validity of current detection results, an independent test using GC-MS was also performed on the same sample containing GD and associated degradation products. The results shown in Figure 5 indicates that both techniques could successfully fingerprint GD. However, the current technique could obtain results within 30 s: this is much faster than the GC-MS technique, which required 8–9 minutes.

### 5.4. Detection of Multiple Chemical Warfare Agents (CWAs)

**Figure 6:**
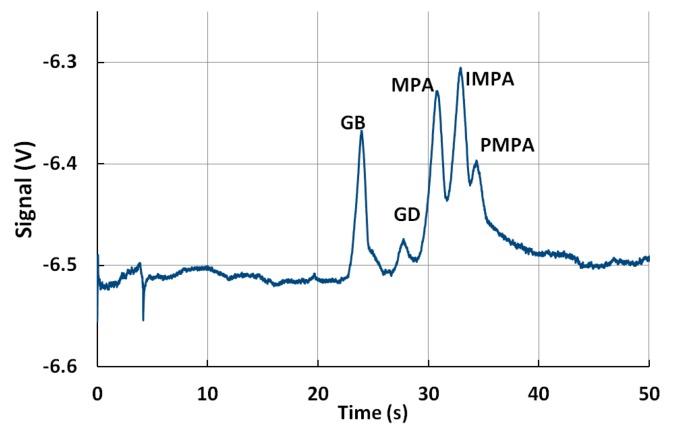
Fingerprinting of GB and GD. Sample (100 ppm GB, GD, MPA, IMPA, PMPA). BGE, 10 mM MOPS, 30 mM Arg, 10 µM CTAB, 30% methanol. Sample injection: 10 s, 1 kV, sample separation: 200 s, 3 kV.

A simultaneous fingerprinting of GB and GD was depicted in Figure 6. A significantly smaller peak could be observed for GD when compared with the same concentration of GB.

GD and its degradation hydrolysis degradation products are detected in less than 30 s. Thirty percent of methanol was used as an organic modifier in CE [53]. The addition of methanol in BGE changes the ratio of hydrodynamic frication to dielectric friction (glycerol has a much lower dielectric friction than water), and could modify the separation efficiency of anions in CE [53]. In comparison with previous tests, the migration time of GB was the fastest (11.37 s) in the BGE of the lowest viscosity, 19.77 s in BGE with 30% of methanol, and slowest in the BGE with the addition of 0.5% of agarose.

### 5.5. Detection of Ricin

**Figure 7:**
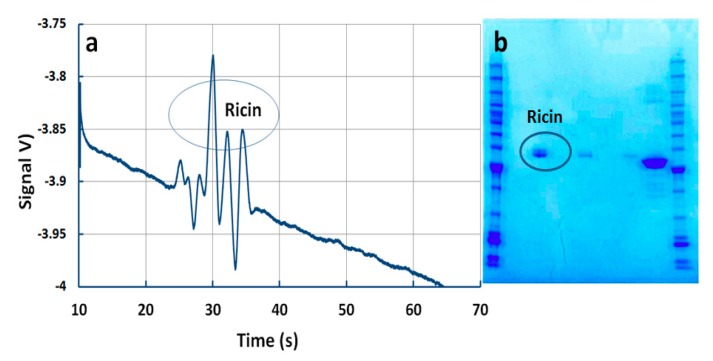
Detection of ricin. (**a**) Detection by current microchip device. Conditions: 2.3 μM ricin in 2.3 M acetic acid + 0.05% Tween 20, (pH = 2.3). Sample injection: 10 s, 1 kV, sample separation: 200 s, 3 kV. (**b**) Detection by PAGE technique.

Ricin is a plant toxin that is present in the seeds of the castor bean plant *Ricinus communis*. It is heterodimetric glycoprotein composed of two subunits, a toxic A subunit (RTA) and a galactose specific lectin B subunit (RTB) via a disulfide bond [44]. Under reducing conditions, it consists of three subunits, i.e., RTA1, RTA2, and RTB [54]. Figure 7a depicts the separation and the detection of these characteristic peaks in less than 25 s. The independent test was performed by DST staff on the same ricin sample using the PAGE gel electrophoresis method (Figure 7b). However, these characteristic peaks could not be revolved, and only one combined peak could be observed (Figure 7b). In addition, PAGE gel electrophoresis required a significantly longer preparation and detection time. To the best of our knowledge, this is the first reporting of the detection of ricin by MCE-$C^{4}D$.

## 6. Conclusions

This paper reports the development of an integrated portable device that could be deployed in the filed for the in situ detection of chemical warfare agents. The device integrates a polymer microchip system, a contactless conductivity detector, a data acquisition and signal processing system, and a graphic/user interface. The raw samples can be loaded onto the microchip cartridge by pipetting. The samples are pre-treated by microchip capillary electrophoresis. The separated analytes are detected by the conductivity-based micro sensors. Chemical warfare agents GB, GD, and VX and their degradation products were tested on the device. The device has also demonstrated the ability to detect ricin molecules and metal ions in water samples. Such devices can potentially be used for the rapid and reliable on-site detection of both chemical and biological agents in the future.

## Figures and Tables

**Table 1:** Reproducibility, linearity, and limit of detection data for the determination of NA hydrolysis degradation products MPA, EMPA, IMPA, and PMPA. MPA: methylphosphonic acid, EMPA: ethylmethylphosphonic acid, IMPA: isopropyl-methylphosphonic acid, PMPA: pinacolylmethylphosphonic acid.

Analyte	Median Relative Migration Time (s)	Relative Migration Time RSD % (n = 120)	Linearity Correlation	Limit of Detection (ppm)
MPA	1.77	0.5	0.9898	1.73
EMPA	1.93	1.1	0.9775	3.73
IMPA	2.12	0.9	0.9775	3.73
PMPA	2.26	2.6	0.9924	4.13
